# Idiopathic membranous nephropathy with renal amyloidosis: A case report

**DOI:** 10.3389/fmed.2022.986065

**Published:** 2022-10-31

**Authors:** Yue Wang, Xueyao Wang, Jinyu Yu, Shan Wu, Zhonggao Xu, Weixia Sun

**Affiliations:** ^1^Department of Nephrology, The First Affiliated Hospital of Jilin University, Changchun, China; ^2^Second Department of Urology, The First Affiliated Hospital of Jilin University, Changchun, China

**Keywords:** idiopathic membranous nephropathy, renal amyloidosis, phospholipase A2 receptor, renal biopsy, case report

## Abstract

**Background:**

Immunoglobulin light chain amyloidosis is a clonal, non-proliferative plasma cell disorder, in which fragments of immunoglobulin light chain are deposited in tissues. Clinical features depend on organs involved but can include restrictive cardiomyopathy, nephrotic syndrome, hepatic failure, peripheral/autonomic neuropathy, and atypical multiple myeloma. Membranous nephropathy (MN) is a group of diseases characterized by deposition of immune complexes under the epithelial cells of glomerular basement and diffuse thickening of the basement membrane. Most patients with idiopathic MN (IMN) have been exposed to phospholipase A2 receptor (PLA2R) antigen, and anti-PLA2R antibodies that attack podocytes can be detected in their blood. IMN combined with amyloidosis nephropathy without secondary factors is rare. The present study describes a patient with IMN combined with immunoglobulin light chain amyloidosis nephropathy.

**Case report:**

A 39-year-old man was admitted to our hospital because of weight loss and edema. His clinical manifestation was nephrotic syndrome. Renal pathology revealed MN. A positive Congo red staining and the pathognomonic apple-green birefringence under cross-polarized light were considered to be associated with amyloid nephropathy. Immunofluorescence showed that λ light chain was positive. Heavy chain deposition disease and amyloid-associated protein amyloidosis were excluded by immunofluorescence and immunohistochemistry, respectively. Subsequent examinations showed that his serum was negative for antibodies against the PLA2R, but PLA2R was present in renal tissue. The final diagnosis was IMN with light chain amyloid nephropathy.

**Conclusion:**

Renal amyloidosis accompanied by IMN is uncommon. Attention should be paid to the subtype of the disease and the exclusion of secondary factors. Perfect clinical and pathological examination are helpful for the classification and staging of the disease. Congo red staining, light microscopy, immunofluorescence, immunohistochemistry, electron microscopic examination, pathological tissue staining for PLA2R antigen and testing for anti-PLA2R antibody in serum are helpful.

## Introduction

Amyloidosis are diseases of protein conformation, caused by misfolding and aggregation of autologous proteins that deposit in tissues as amyloid fibrils. Amyloid immunoglobulin light chain (AL) amyloidosis is characterized by a clonal population of bone marrow plasma cells that produce a monoclonal kappa (κ) or lambda (λ) light chain (1), with determination that amyloid is composed of immunoglobulin light chains required for diagnosis (2). More than 75% of patients with systemic amyloidosis patients have AL amyloidosis, with about 65% of the latter showing kidney involvement (3). The primary manifestation of renal amyloidosis is the deposition of amyloid substances in the glomeruli, which may be accompanied by deposition in the renal interstitium and vascular wall.

Membranous nephropathy (MN) is an autoimmune disease caused by the deposition of immune complexes under epithelial cells of glomerular basement. Podocytes in these patients are specifically attacked by antibodies against phospholipase A2 receptor (PLA2R). The pathogeneses of idiopathic MN (IMN) and amyloidosis nephropathy are different (4). The present study describes a patient who presented with nephrotic syndrome (NS), which was subsequently diagnosed as IMN with renal amyloidosis.

## Case presentation

A 39-year-old Chinese man was admitted to our nephropathy department with weight loss for the previous 1 year and edema for 40 days in April 2018. His appetite was poor and his consumption of meat was reduced. Edema was observed in both lower extremities. He was diagnosed at a local hospital with hypoproteinemia and proteinuria, and was prescribed cephalosporin and a diuretic, but there was no obvious improvement. He had no history of kidney disease, no history of other systemic or genetic diseases and had no psychosocial history. During the course of the disease, he experienced occasional flustering and bloating. His daily urine volume was about 600 ml. His weight had dropped 10–15 kg during the previous year. Physical examination showed that his blood pressure was 99/68 mmHg, he had a bulging abdomen, and he had severe edema in his lower extremities. Other results of physical examinations were within normal limits.

Laboratory parameters at the time of kidney biopsy are shown in Table 1. His serum creatinine was 95.3 μmol/L and serum albumin was 13.1 g/L. The daily urine protein excretion was 8.01 g/24 h, the daily urinary secretion of κ light chain was 90.10 mg/24 h, and λ light chain was 141.10 mg/24 h. The serum free-κ and free-λ chains were 27.10 mg/L and 145.00 mg/L, respectively. An electrocardiogram indicated low and inverted T waves. The chest computed tomography (CT) showed bronchitis, slight inflammation of the lungs, slight enlargement of the mediastinal and right hilar lymph nodes, coronary calcification, and ascites. Urinary color Doppler ultrasound suggested enhanced echogenicity of the renal cortex and ascites, and that the maximum depth in the dark region of the lower abdomen was 100 mm. Color Doppler echocardiography showed that there were no obvious abnormalities in cardiac structure and blood flow. No obvious abnormalities were found in other laboratory indicators (see Table 1 for details). Hepatitis B virus, hepatitis C virus, human immunodeficiency virus and syphilis antibody were negative.

**TABLE 1:** Laboratory parameters at the time of kidney biopsy.

Laboratory parameter	Value	Units	Normal range	Finding
Platelets	450	10^9^/L	125–350	High
Hemoglobin	171	g/L	130–175	Normal
γ-Glutamyl transpeptidase	226.8	U/L	10.0–60.0	High
Cholinesterase	12,658	U/L	4,300–12,000	High
Total protein	42.1	g/L	65.0–85.0	Low
Serum albumin	13.1	g/L	40.0–55.0	Low
Albumin/Globulin ratio	0.45		1.2–2.4	Low
Blood urea nitrogen	5.36	mmol/L	3.1–8.0	Normal
Creatinine	95.3	μmol/L	57–97	Normal
Blood uric acid	532	μmol/L	208–428	High
Cystatin C	1.31	mg/L	0.38–1.26	High
Cholesterol	19.29	mmol/L	2.6–6.0	High
Triglyceride	4.98	mmol/L	0.28–1.80	High
Low density lipoprotein cholesterol	11.36	mmol/L	2.06–3.10	High
Blood calcium	1.94	mmol/L	2.11–2.52	Low
Urinary proteinuria	3 +		NA	High
Urinary sediments red blood cell	13.1	/HPF	0.0–3.0	High
Urine cast	46	/LPF	0–2	High
Urinary protein excretion	8.01	g/24 h	<0.20	High
Urine microalbumin	6570.50	mg/24 h	0–60	High
Urine α1 microglobulin	70.64	mg/24 h	<24	High
Urine β2 microglobulin	0.4	mg/24 h	<0.4	High
Urine IgG	260.95	mg/24 h	0–17.0	High
Urine free κ light chain	90.10	mg/24 h	<14.20	High
Urine free λ light chain	141.10	mg/24 h	<7.80	High
Thyroid stimulating hormone	20.000	uIU/ml	0.27–4.2	High
Free triiodothyronine	2.96	pmol/L	3.1–6.8	High
Free thyroxine	8.49	pmol/L	12.0–22.0	High
Serum IgG	3.06	g/L	7.0–16.0	Low
Serum IgA	3.47	g/L	0.7–4.0	Normal
Serum IgM	2.50	g/L	0.4–2.3	High
C3	1.80	g/L	0.9–1.8	Normal
C4	0.75	g/L	0.1–0.4	High
Erythrocyte sedimentation rate	47	mm/1 h	0–15	High
Serum protein electrophoresis	Normal		NA	Normal
Urine immunofixation electrophoresis	Normal		NA	Normal
Blood immunofixation electrophoresis	Normal		NA	Normal
Serum free-κ chain	27.10	mg/L	6.7–22.4	High
Serum free-λ chain	145.00	mg/L	8.3–27.00	High
Brain natriuretic polypeptide	264.0	pg/ml	0–125	High
Myoglobin	41.7	ng/ml	0–121	Normal
Creatine kinase- myoglobin	0.88	ng/ml	0–3.38	Normal
Cardiac troponin I	0.020	ng/ml	0–0.034	Normal
Carbohydrate antigen 125	459.86	U/ml	<35.00	High
Neuron specific enolase	34.06	ng/ml	<25.00	High

**FIGURE 1:**
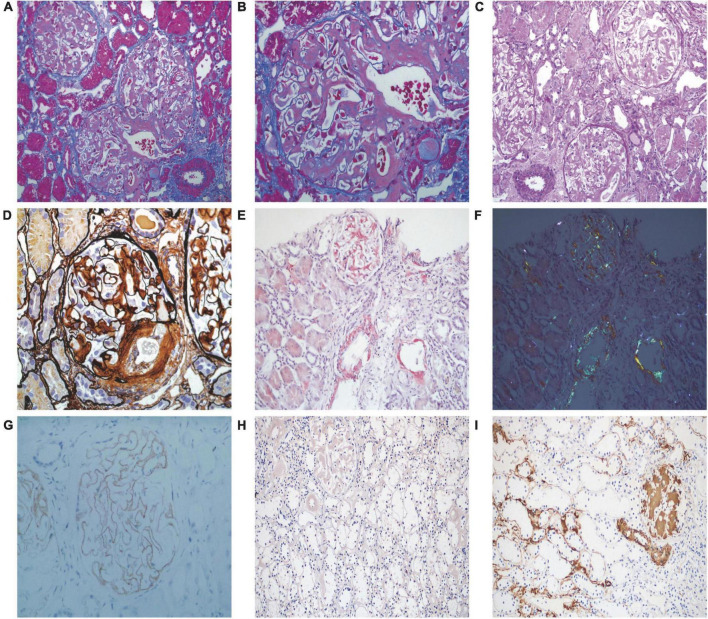
Pathological features of light microscopy and immunohistochemistry. **(A,B)** Small amounts of erythrophilin were deposited under epithelial cells, and vacuolar degeneration of renal tubular epithelial cells (Masson staining, 200 × and 400×, respectively). **(C)** Deposition of eosinophilic homogeneous unstructured material in the mesangial region, capillary wall, local interstitium, and some arteriolar walls (PAS staining, 200×). **(D)** Diffused mesangial broadening, with the basement membrane thickened segmentally, no absence of spike formation and no eyelash-like changes in the glomerular basement membrane (PASM staining, 400×). **(E)** Positive staining of Congo red of glomeruli, partial arteriole walls, and local interstitium (Congo red staining, 200×). **(F)** Pathological apple-green birefringence under cross-polarized light (200×). **(G)** Fine granular deposition of phospholipase A2 receptor along the capillary walls (immunohistochemistry, 400×). **(H)** Amyloid-associated protein was negative in the mesangial area of the glomeruli and arteriolar wall, as well as in the focal renal interstitium (immunohistochemistry, 200×). **(I)** Amyloid-associated protein staining positive control (immunohistochemistry, 200×).

**FIGURE 2:**
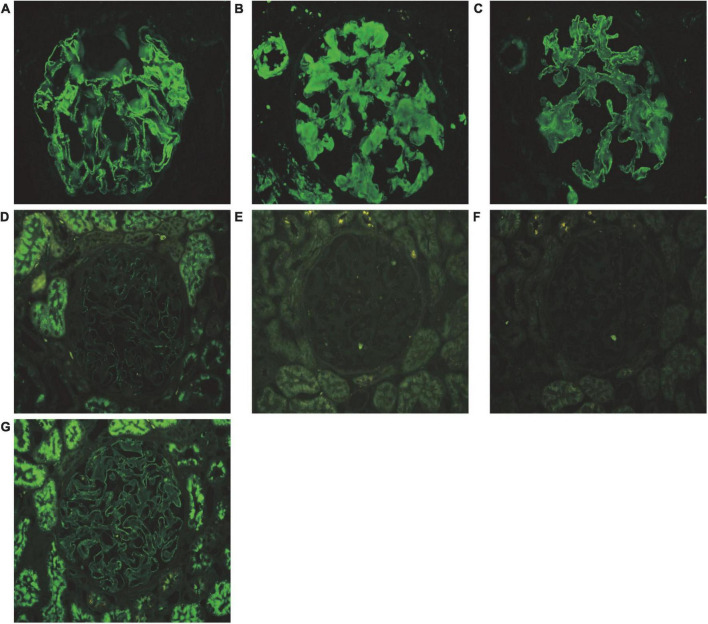
Pathological features of immunofluorescence. **(A)** Granular deposition of IgG (+++) along the glomerular capillary walls (immunofluorescence, 400×). **(B)** Deposits of λ (++) along the arterioles, mesangial glomeruli, and capillary walls (immunofluorescence, 400×). **(C)** Deposits of κ (+) along the glomerular capillary walls (immunofluorescence, 400×). **(D)** Weak positive of IgG1 deposition in the glomerular basement membrane (immunofluorescence, 400×). **(E)** IgG2 was negative in the glomerular basement membrane (immunofluorescence, 400×). **(F)** IgG3 was negative in the glomerular basement membrane (immunofluorescence, 400×). **(G)** Positive of IgG4 deposition in the glomerular basement membrane (immunofluorescence, 400×). **(A–C)** Show the immunofluorescence of frozen section, and **(D–G)** show the immunofluorescence of paraffin section.

**FIGURE 3:**
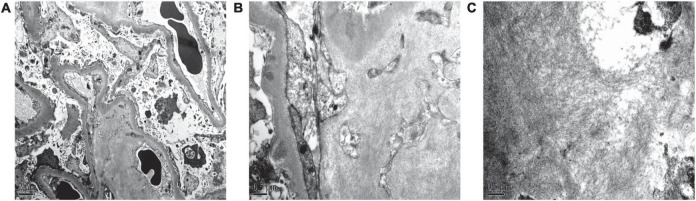
Renal pathological features of electron microscopy. **(A)** Massive electron dense deposition in the subepithelial and basement membranes, and extensive fusion of epithelial podocytes (electron microscopy, 6,000×). **(B,C)** Scattered fibrous deposits in the mesangial region, basement membrane, and interstitium of the kidney (electron microscopy, 25,000× and 30,000×, respectively).

Renal biopsy was performed on May 2, 2018. Light microscopy showed 38 glomeruli, one was spherical sclerosis, whereas in the remaining glomeruli, there was small amounts of erythrophilin deposition under the epithelial cells (Figures 1A,B). The glomeruli had diffused mesangial broadening, and an eosinophilic homogeneous non-structural substance was found to be deposited diffusely in mesangial areas and capillary walls (Figure 1C). The basement membrane thickened segmentally, but there were no absence of spike formation and no eyelash structure in the glomerular basement membrane (GBM) (Figure 1D). Large numbers of lymphocytes and macrophages were found to have infiltrated in renal interstitium focally, accompanied by slight fibrosis. Small amounts of eosinophilic homogeneous non-structural deposits were observed in the interstitium and in some arteriole walls, with slight thickening of some arterioles (Figure 1C). Congo red staining showed that the glomeruli, partial arteriole wall and local interstitium were positive (Figure 1E). Pathological apple-green birefringence was produced under cross-polarized light (Figure 1F). Immunohistochemistry showed positive expression fine granular deposition of PLA2R along the capillary walls (Figure 1G). It also showed positivity for λ and slight positivity for κ. Immunohistochemical staining of paraffin sections showed that amyloid-associated protein (AA) was negative in the mesangial area of the glomeruli and arteriolar wall, as well as in the focal renal interstitium (Figures 1H,I is the positive control). Immunofluorescence showed granular deposition along the glomerular capillary wall with very strongly positive for IgG (Figure 2A), strongly positive for IgM, slightly positive for IgA, and negative for C3, C4, C1q, and fibrinogen. The small arteries, glomerular mesangial area, and capillary walls were strongly positive for λ (Figure 2B), with κ deposits observed along the glomerular capillary walls (Figure 2C). Immunofluorescence of IgG subtypes in paraffin sections showed that IgG1 weak positive (Figure 2D), IgG2 and IgG3 negative (Figures 2E,F), IgG4 positive deposit in the GBM (Figure 2G). Electron microscopy indicated that massive electron dense deposition in the glomerular subepithelial and GBM, and extensive fusion of epithelial podocytes (Figure 3A). It also showed the absence of cellular broadening in the GBM and the mesangial area of the glomeruli, along with disorderly deposits of fibrous material (Figures 3B,C). The renal interstitium had the same characteristic deposition of fibrous material (Figure 3B).

Based on the clinical indicators and pathological findings, this patient was finally diagnosed with NS, with the pathology type being IMN with AL type amyloid nephropathy. The patient also had hyperuricemia, peritoneal effusion, and hypothyroidism. The chest CT showed slight inflammatory changes, but the patient had no respiratory tract infection symptoms, and the infection-related indicators were normal. Therefore, we considered the patient did not have infection or chronic inflammation. The patient had hypotension, but no palpitation. Examination revealed sinus rhythm and normal cardiac structure and blood flow. Therefore, we considered the hypotension might be related to amyloidosis. The patient had a large amount of ascites, but the liver function of the patient was normal. Hypoproteinemia caused by NS can cause edema, and the decrease in daily urine volume will aggravate it. Therefore, we believed that the cause of ascites was leakage due to hypoproteinemia caused by NS.

Treatment was primarily aimed at preventing the complications of NS. While hospitalized, the patient was treated with statins to control blood lipid concentrations, aspirin as an anticoagulant, and intermittent intravenous supplementation of human serum albumin and diuretics to promote urination and reduce edema. He was also treated with thyroxine to improve his thyroid gland hypofunction. The patient was advised to go to the Hematology Department for further bone marrow biopsy and to determine the treatment plan for amyloidosis, but he refused for financial reasons. After discharge on May 5, 2018, the patient was not regularly followed up in our hospital, and was lost to follow-up.

## Discussion

This patient was a middle-aged male with chronic onset of disease. He had no history of other medical diseases. Clinical manifestations included NS and mildly elevated serum creatinine. His weight had decreased significantly in 1 year. Besides renal damage, the patient also had hypotension, which made us suspect he was likely to have secondary NS. Proteinuria classification showed a significant increase in urine λ-light chain and a concomitant increase in serum free-λ chain. Renal puncture biopsy showed the glomeruli, some arteriolar walls, and the local interstitium were positively stained with Congo red, and pathological apple-green birefringence was produced under cross-polarized light. Immunofluorescence suggested strongly positive deposition of λ chain along the small arteries, glomerular mesangial area and capillary walls, but κ chain deposition was weak. IgG, IgM were deposited in the glomerular capillary wall in granular form, but not in the area of amyloid deposition, and IgA was (±), so we did not consider the deposition of heavy chain in the deposition area of amyloid. Immunohistochemistry showed that AA was negative, so AA amyloidosis could be ruled out. Electron microscopy showed an absence of cellular broadening in the mesangial region and basement membrane of the glomeruli, with scattered fibrous deposits in the mesangial region and renal interstitium. Renal pathological examination indicated that the deposition area of amyloid was consistent with the deposition area of λ. The above pathological changes suggested amyloid nephropathy, and the subtype was AL type.

However, to our surprise, the renal pathology also had the characteristics of MN. Light microscopy showed segmental thickening of the GBM. Immunofluorescence showed granular deposition of IgG along the glomerular capillary walls. Electron microscopy showed a large amount of electron dense deposits in the subepithelial membrane and GBM, and extensive fusion of epithelial podocytes. These typical pathology findings suggest that the patient’s renal pathology can be diagnosed as MN. In order to further clarify whether the patient is an IMN, we tested the serum anti-PLA2R antibody IgG and the result was negative. Therefore, we further detected PLA2R in renal biopsies. Immunohistochemistry showed that PLA2R was positively expressed along the fine particle deposition of capillary walls. Based on the medical history and clinical auxiliary examination, diagnoses of secondary MN were excluded. The above information supported the patient with IMN.

Amyloidosis is a group of diseases caused by the deposition of amyloid protein in the extracellular matrix, resulting in tissue and organ damage at the deposition site. The kidneys are most frequently involved in systemic amyloidosis, with renal amyloidosis in most patients due to immune globulin (5). AL type amyloidosis is the most common type of systemic amyloidosis and has been associated with the abnormal proliferation of monoclonal plasma cells, as well as with lymphoproliferative diseases (6). According to the precursor proteins that form amyloid fibroids, amyloidosis can be divided into primary systemic amyloidosis such as AL type and amyloid immunoglobulin heavy chain type, secondary systemic amyloidosis also known as AA type, hereditary amyloidosis, and other main types (7). Patients with amyloidosis are staged using the Mayo Clinic staging system (8).

The incidence of amyloidosis is uncertain, but AL amyloidosis is thought to have an annual incidence of 6–10 per million persons in the United Kingdom and United States (7). Although the incidence rate in China is undetermined, domestic renal biopsy data have found that AL amyloidosis is present in about 3.63% of patients with secondary kidney disease (9). In developed countries, approximately 75–80% of all MN are idiopathic, the remaining 20–25% are secondary to different conditions (10, 11). In recent years, in China, the incidence rate of MN has also shown a trend of gradually increasing (9). But MN with amyloid nephropathy is rare. MN complicated with renal amyloidosis has been reported abroad, but most of them are secondary MN complicated with amyloidosis, such as chronic lymphocytic leukemia, rheumatoid arthritis and Waldenström’s macroglobulinemia, which may lead to the deposition of immune complexes in glomeruli caused by antigen exposure (12–14). Four cases of amyloid nephropathy complicated with IMN have been reported in China (15–18). The renal amyloidosis and IMN mentioned in the above four cases are considered to be separate diseases without other secondary factors.

Our case has its own characteristics compared with the above-mentioned reports of renal amyloidosis with IMN. In terms of clinical indicators, all the above cases (except one case that was not tested) were positive for serum anti-PLA2R antibody. But in our case, the patient’s serum anti-PLA2R antibodies was negative, kidney tissue PLA2R antigen was positive, the reasons might be as follows: (1) 70% of patients with IMN had positive circulating anti-PLA2R autoantibodies (4). Circulating serum anti-PLA2R antibodies may be negative in some situation, such as when the time from the onset of MN to renal biopsy is too long, during disease immune remission, when the affinity between antigen and antibody is strong, or when some other reasons lead to fast antibody clearance (19). (2) It may be related to the imperfect detection technology. (3) This patient had amyloid nephropathy, but it was not excluded that abnormal light chain synthesis by plasma cells affected the production of antibodies against PLA2R by B cells. When the serum anti-PLA2R antibodies is negative, the PLA2R antigen can still be detected in the kidney, indicating that it has a higher sensitivity. Many studies in recent years have shown that serum anti-PLA2R antibodies were closely related to IMN disease activity, and suggested that the change of the anti-PLA2R antibody level in IMN patients is closely related to the status of IMN (20–22). According to the latest KIDIGO guidelines (23), a kidney biopsy may not be needed in anti-PLA2R-positive patients with a low risk of disease progression and/or a high risk of biopsy-related morbidity. In serum anti-PLA2R antibody negative patients, a kidney biopsy is needed to diagnose MN. In such patients, it is important to look at whether PLA2R staining is present in the glomeruli, because this will allow identification of patients with PLA2R-associated MN.

In terms of renal pathology, IMN in all of the above cases (except one case not described) was characterized by spike formation. But in our case, the pathological Periodic acid-silver methenamine staining did not show the formation of spike due to deposition of immune complexes and lash-like structural changes due to amyloid of the GBM. We consider the absence of spike formation is related to the simultaneous deposition of amyloid in GBM. Amyloid is mainly deposited in the mesangial region of the glomeruli and the basement membrane of capillaries, the basement membrane of renal tubules and the walls of arterioles. In severe cases, amyloid can be deposited in the renal interstitium. At the early stage, the GBM was slightly thickened, with Periodic acid-silver methenamine staining showing segmental eyelash structures as a result of amyloid deposition under the GBM epithelium (24, 25), it can be easily confused with MN. According to the characteristics of amyloid protein under electron microscopy and the immunofluorescence characteristics of MN, the two diseases could be distinguished. Glomerular damage in amyloidosis usually occurs without GBM thickening. Segmental elongated “spicules” extending from the GBM are a common feature (5, 26). There were cases reported that non-branching fibrils could focally appeared to disrupt the GBM and protrude into Bowman space into the cytoplasm of the podocytes. Podocyte foot processes were extensively effaced. Pathological findings of these above two cases showed amyloid nephropathy with obvious epithelial hyperplasia and glomerular collapse with clinical manifestations of acute kidney injury and NS (27). There is no known case of amyloidosis leading to exposure of GBM antigens and antibodies production leading to MN. It is not clear whether IMN is caused by antigen exposure after amyloid deposition in this case. However, considering the absence of disruption of GBM due to amyloidosis in this case, and in combination with other pathological findings, it is unlikely that IMN is caused by amyloidosis, we considered renal amyloidosis and MN as independent of each other.

The diagnostic criteria of MN and the diagnosis and treatment of AL amyloidosis criteria have been defined according to the KDIGO guidelines published in 2020 (23) and the guidelines for the diagnosis and treatment of AL amyloidosis developed by the Standards Committee of the British Society of Hematology (28). B-cell anomalies play a role in the pathogenesis of MN. Selective B-cell removal by rituximab appears to be a more promising treatment compared with cyclophosphamide, which has the characteristic of non-selective B-cell depletion (29). 2020 KDIGO guidelines for glomerulonephritis suggest that (23), for patients with MN and at least one risk factor for disease progression, using rituximab or cyclophosphamide and steroids for six months is recommended. Rituximab has become a first-line treatment in the treatment of MN. The treatment of patients with AL amyloidosis is based on the treatment of multiple myeloma, but there is no standard treatment for the former. Localized amyloidosis can be treated by local resection or radiation (28). Other treatments can include autologous peripheral blood stem cell transplantation; anti-plasma cell therapy, such as proteasome inhibitor, monoclonal antibodies, immunomodulatory drugs, and alkylating agents; anti-amyloid filament therapy; and supportive therapy (6, 28, 30, 31). Daratumumab is a humanized monoclonal IgG1-κ antibody that targets the plasma cell surface CD38 antigen (6, 30). The ANDROMEDA trial showed that subcutaneous injection of cyclophosphamide, bortezomib, dexamethasone combined with daratumumab improved overall response rate, organ response rate, and progression-free survival time of major organs (32). Doxycycline was found to interfere with amyloid fibril formation in a transgenic mouse model of AL amyloidosis (33), suggesting that doxycycline in combination with other agents can be used during the first year after diagnosis to treat patients with AL amyloidosis who are or are not transplant eligible (6, 30).

CD20 or CD38 may be the main source of autoantibodies in IMN. For patients with high anti-PLA2R antibody titers, reducing CD20/38 may be an effective intervention (34, 35). At the same time, CD38 is also an ideal target for the treatment of amyloidosis (36). A multinational research team from the United States and Belgium published the latest research on felzartamab, which proved it can effectively reduce the anti-PLA2R antibody titer of patients with MN, and felzartamab, as an anti-CD38 monoclonal antibody, has good therapeutic potential in the treatment of light chain amyloidosis (37). This may provide a new therapeutic prospect for IMN with amyloid nephropathy patients.

## Conclusion

The combination of MN and amyloidosis is rare. This study described a patient with IMN accompanied with amyloid nephropathy. This case has some shortcomings. The patient did not undergo bone marrow biopsy and accept drug treatment in our hospital. He did not return to our hospital for follow-up and he was lost to follow-up, which made the case incomplete. However, we hope to deepen our understanding of this disease through the sharing of this case. The detection of serum anti-PLA2R antibody and glomerular PLA2R antigen is helpful for the diagnosis of IMN. In addition to light microscopy, Congo red staining and immunofluorescence examination, since early AL renal amyloidosis may not have obvious light microscopy and immunopathological features, electron microscopy is very important for the diagnosis of renal amyloidosis. For cases with unclear classification, immunohistochemistry, immunoelectron microscopy, mass spectrometry and even genetic testing can be used if necessary.

## Data availability statement

The original contributions presented in this study are included in the article/supplementary material, further inquiries can be directed to the corresponding author.

## Ethics statement

Written informed consent was obtained from the individual(s) for the publication of any potentially identifiable images or data included in this article.

## Author contributions

YW, XW, and WS collected, analyzed, and reviewed the clinical data of the patient. JY and SW performed and diagnosed the histological examinations of the kidney biopsy sample. WS and ZX suggested the revisions of the manuscript. All authors read and approved the final manuscript.

## Abbreviations

MN: Membranous nephropathy
IMN: Idiopathic membranous nephropathy
PLA2R: Phospholipase A2 receptor
AL: Amyloid immunoglobulin light chain
κ: Kappa
λ: Lambda
NS: Nephrotic syndrome
CT: Computed tomography
GBM: Glomerular basement membrane
AA: Amyloid-associated protein.
